# Evaluation and Management of Premature Rupture of Membranes: A Review Article

**DOI:** 10.7759/cureus.36615

**Published:** 2023-03-24

**Authors:** Aditi Garg, Arpita Jaiswal

**Affiliations:** 1 Department of Obstetrics and Gynaecology, Datta Meghe Institute of Higher Education and Research, Jawaharlal Nehru Medical College, Wardha, IND

**Keywords:** prom, corticosteroid in prom, antibiotic therapy in prom, amnisure test, amnio dye test, nitrazine test, preterm prom, chorioamnionitis, prom diagnosis, prematurity

## Abstract

Premature rupture of membranes (PROM), now also referred to as "pre-labour rupture of membranes," is the rupture of gestational membranes after 37 weeks but before the process of labour begins. When membrane rupture occurs before 37 weeks of gestation, it is referred to as preterm PROM (PPROM). Prematurity is held accountable for the majority of newborn morbidity and mortality. PROM causes around one-third of all preterm deliveries and complicates 3% of pregnancies. Significant morbidity and mortality rates have been associated with PROM. Preterm (PROM) pregnancies are more difficult to manage. Pre-labour rupture of membranes is characterised by its short latency, higher intrauterine infection risk, and greater umbilical cord compression probability. Women with preterm PROM are more likely to develop chorioamnionitis and placental abruption. Various diagnostic modalities include sterile speculum examination, the nitrazine test, the ferning test, and the latest advances, which are the Amnisure test and the Actim test. Despite all these tests, there is still a need for newer, non-invasive, rapid, and accurate tests. Admission to a hospital, amniocentesis to rule out infection, and, if necessary, prenatal corticosteroids and broad-spectrum antibiotics are all alternatives for treatment. As a result, the clinician managing a pregnant woman whose pregnancy has been affected by PROM plays a crucial role in the management and must be well aware of probable complications and control measures to reduce risks and increase the likelihood of the required outcome. PROM's proclivity for recurrence in later pregnancies provides a chance for prevention. Furthermore, prenatal and neonatal care developments will continue to enhance the outcomes of women and their children. The purpose of this article is to summarise the concepts related to the evaluation and management of PROM.

## Introduction and background

The inner aspect of the gravid intrauterine cavity is lined by the membranes of the foetus, which are referred to as the placental membranes or the amniochorionic membranes. These foetal tissues separate two compartments, namely the maternal and fetoplacental compartments. Foetal membranes consist of the amnion, the deepest layer of the intraamniotic cavity, and the chorion, which connects to the maternal decidua to form the placental tissue. A collagen-rich extracellular matrix connects the amnion and chorion [1]. Typically, membranes rupture during the course of labour, i.e., at full dilatation of the cervix. Pre-labour rupture of membranes occurs when the membranes rupture after 37 weeks but before the process of labour begins. Preterm PROM is defined as the rupture of membranes before 37 weeks of pregnancy. Pre-labour rupture of membranes is a significant obstetric problem often ignored and occurs in about 3%-4% of all pregnancies. It contributes 40% to 50% of all preterm births [2,3]. In comparison to preeclampsia and gestational diabetes, the incidence of PROM is much higher. Preterm PROM contributes more significantly to neonatal mortality and morbidity than any other group of disorders [1]. Despite significant advances in prenatal treatment during the last three decades, rates of PROM and associated preterm births have increased. This review article aims to analyse and explain the concepts related to PROM, focusing mainly on its evaluation and diagnosis.

## Review

Methodology

**Figure 1:**
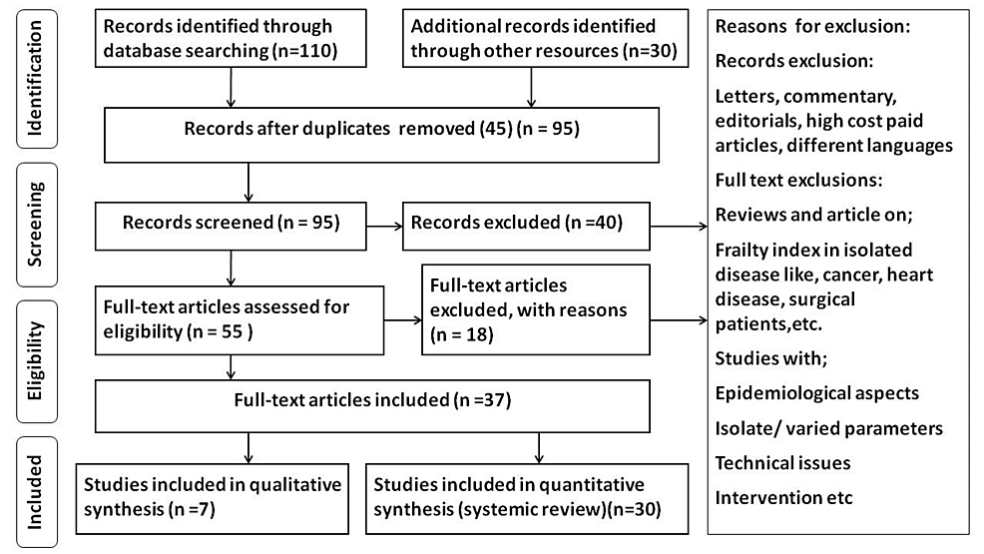
PRISMA model for search strategy

We used PubMed to search Medline and the Cochrane Central Register of Controlled Trials (CENTRAL) databases through the Cochrane Library. The search strategy for PubMed was tailored to individual databases. It was as follows: "premature rupture of membrane" (Title/Abstract) AND "chorioamnionitis" (Title/Abstract) OR "nitrazine test" (Title/Abstract) OR "amnisure test" (Title/Abstract) OR "preterm PROM" (Title/Abstract). Additionally, we looked for other studies by screening the references list for potentially pertinent articles. Studies that were found through these electronic searches, as well as relevant sources included in their bibliographies, were examined. Original studies in English that assessed the risk factors, diagnosis, and management were included. The Preferred Reporting Items for Systematic Reviews and Meta-Analyses (PRISMA) research approach is displayed in Figure 1.

Risk factors

**Figure 2:**
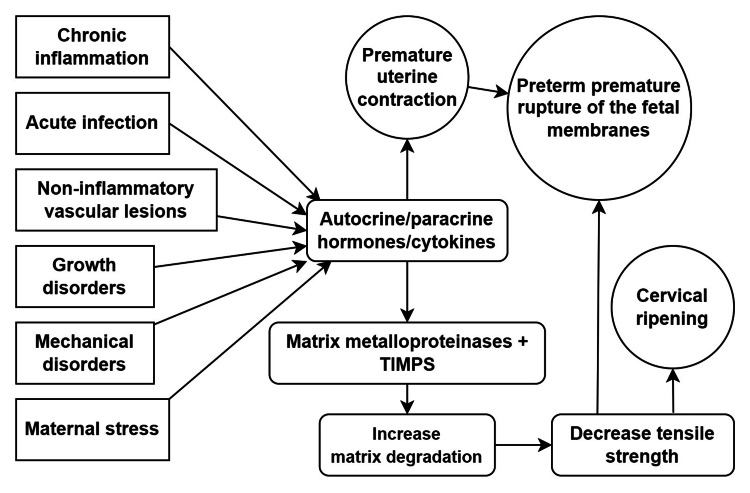
Risk factors and etiopathogenesis of premature rupture of membranes TIMPS: Tissue inhibitors of metalloproteinases; factors like chronic inflammation, acute infections, non-inflammatory vascular lesions, growth disorders, mechanical disorders, and maternal stress lead to the activation of hormones and cytokines, which eventually increase matrix degradation and cause premature uterine contractions, ultimately leading to premature rupture of membranes [4-7].

Assessment of risk factors remains the mainstay in the evaluation of pre-labour rupture of membranes. The best way to avoid PROM problems is to predict and eliminate the risk factors that cause them. The risk factors for PROM include preterm birth in a previous pregnancy, addiction such as cigarette smoking, polyhydramnios, urinary and sexually transmitted infections, chronic steroid use, twin pregnancy, last PROM, antepartum vaginal bleeding, direct abdominal trauma, collagen vascular disorders like Ehler Danlos and systemic lupus erythematosus (SLE), low basal metabolic rate (BMI), anaemia, and low socioeconomic status [4]. Specific invasive techniques can damage the membranes, causing them to leak. Amniocentesis, chorionic sampling and cervical cerclage are uncommon causes of preterm PROM [4]. The recurrence rate of preterm PROM is 16% to 32% compared to about 4% in women who have had a previous term delivery [5-7]. The risk factors and etiopathogenesis of premature rupture of membranes are shown in Figure 2.

Evaluation

The first step in patient evaluation is confirmation of the diagnosis. History taking and physical examination form the mainstay of diagnosis. A pregnant woman presenting with a history of sudden gush of fluid from the vagina leading to soaking of her clothes must warrant suspicion of PROM or PPROM. The fluid is described as either clear or pale yellow by the patient. The first step in the examination is sterile per speculum examination. The diagnosis is usually confirmed on a sterile speculum examination in a patient complaining of "watery vaginal discharge". It depends on the obstetrician's skills to identify the three clinical signs. These are 1) the pooling of fluid in the vaginal vault or fluid leaking from the cervical os; 2) the nitrazine paper test: if it turns blue, it is suggestive of alkaline fluid (amniotic fluid); 3) the fern test: a sample of amniotic fluid is fixed on the slide and observed under the microscope. Amniotic fluid appears as a fern-like pattern.

Considering diagnostic validity, cost, and technical simplicity, all of these clinical indicators have certain limitations [4]. On a daily basis, the commonly used test for the diagnosis of PROM is based on the alkaline ph of the cervicovaginal secretions, called the nitrazine test. Although the test carries a high rate of false positives, it is still the most common test in diagnosing PROM. The fern test also has a high chance of producing false positives and false negatives. It is based on the microscopic crystallisation of amniotic fluid on drying, which is usually collected with a sterile swab from the posterior fornix of the vagina. The false negatives are mainly due to technical error or contamination with blood, whereas false positives are due to contamination with cervical mucus or semen and fingerprints. The fern test's reported sensitivity and specificity in patients without labour are 51% and 70%, respectively, and 98% and 88%, respectively, in patients in labour [5]. Only when conventional tests for PROM do not give a confirmatory diagnosis are alternative tests used, such as the amnio-dye test. Indigo carmine is the most commonly used dye for infusion into the amniotic cavity, and the leakage of blue fluid per vagina is confirmatory [6]. The amnio-dye test also comes with some intrinsic risks of placental abruption leading to bleeding, spontaneous abortion, infection, and sepsis as it is an invasive procedure [4]. Though many researchers may consider it the gold standard for diagnosis, there is still an immediate need for another non-invasive, precise, and affordable test.

Some nonspecific marker tests that are used less commonly for diagnosing membrane rupture are fetal fibronectin, β-human chorionic gonadotropin (β-hcg) [7-11], alpha-fetoprotein [8,9,12], and placental alpha microglobulin 1 (PAMG1) [13-16]. However, it has been shown that these markers indicate more decidual disruption than membrane rupture and hence are not of much value [14,15]. Immediate labour induction is required in women suffering from complications like chorioamnionitis, bleeding due to placental abruption, and fetal distress [6]. A recent advance in the diagnosis of PROM is the amnisure test [13-16]. Amnisure immunoassay is straightforward, simple to use, quick, and non-invasive. Placental alpha microglobulin 1 is a glycoprotein that has a placental origin and is prevalent in amniotic fluid, but its proportion in maternal blood is deficient. It can be detected even in trace amounts by this test. The concentration of this protein in the cervicovaginal secretions is very low and is even lower when the membranes are intact. Higher concentrations of PAMG1 in amniotic fluid make it a reliable marker for diagnosis [4]. The newly discovered Actim PROM test for insulin-like growth factor binding protein 1 (IGFBP-1) detection in the vaginal fluid is a simple bedside test that may be used as a supplement to the clinical diagnosis of PROM. A single line in the slide indicates that the test is negative for amniotic fluid, and a double line indicates a positive result [17,18]. According to studies, it is considered more sensitive and specific than the Amnisure test [19]. Those with intact membranes are essentially excluded by a negative test result [20]. It has been proposed that if a patient tests negative, further c-reactive protein (CRP) and white blood cell (WBC) counts must be done to exclude infections [21]. A downside, however, is that due to membrane stretching, IGFBP-1 levels rise in the cervicovaginal secretions of pregnant women with imminent preterm labour and ripening cervix even in the absence of ruptured membranes [22,23].

Management

Expectant Management

According to recent guidelines, expectant management is done in cases of preterm (<34 weeks) and late preterm (34-36 weeks + six days) PROM if no contraindications exist. It consists of admitting the patient, administering a course of corticosteroids, short-term tocolysis, rectovaginal swabs for group-B streptococci screening, and starting the group-B streptococci prophylaxis. Antibiotics are stopped if the screening is negative. Magnesium sulphate is administered for neuroprotection if the gestational age is less than 32 weeks. Management of PPROM between 34-37 weeks + six days is the same as that of <34 weeks, except that tocolytics are not administered, and induction of labour is performed if there are indications for the same [24]. Monitoring maternal vitals is of paramount importance to rule out the presence of chorioamnionitis. It is a clinical diagnosis suggested by the presence of fever, tachycardia, and/or uterine tenderness. A speculum examination can also confirm the diagnosis if it shows the presence of pus leaking from the cervix. Maternal monitoring is done for temperature, uterine tenderness, and uterine contractions. Foetal monitoring is done by a daily non-stress test (NST) and biophysical score if the NST is non-reactive. Periodic USG is done to monitor foetal growth. An elevated WBC count, elevated lactate dehydrogenase (LDH), and reduced glucose seen on amniotic fluid sampling may also point towards the diagnosis. In such cases, delivery of the foetus is initiated immediately, followed by the administration of broad-spectrum antibiotic therapy [4]. 1059 pregnancies complicated by preterm PROM and handled expectantly have been documented in the recent two decades. Overall, 51.8% of neonates survived. Pulmonary hypoplasia and skeletal abnormalities were both rare occurrences [25].

Antibiotic Regime in the Case of PROM

Antibiotics have become the mainstay of therapy for patients with pre-labour membrane rupture. However, little is known about the natural course of intrauterine infection during antibiotic therapy [25]. Studies have shown that bacterial contamination of amniotic fluid can occur because of amniocentesis. Life-threatening complications like Neonatal intraventricular bleeding, white brain matter injury, bronchopulmonary dysplasia (BPD), necrotizing enterocolitis (NEC), and sepsis can occur as a consequence of chorioamnionitis; hence the role of antibiotics is prime importance [26]. Two major trials were carried out to evaluate the effectiveness of antibiotic use in preterm PROM. These included the Eunice Kennedy Shriver National Institute of Child Health and Human Development (NICHD)-Maternal-Fetal Medicine Units (MFMU) study along with the ORACLE trial [27,28]. Erythromycin, amoxiclav, or amoxiclav in conjunction with erythromycin were utilised in the ORACLE experiment. Their findings were different in that there was no meaningful improvement in delivery latency (less than seven days). As expected in the primary outcome, infant morbidity was not decreased (death, chronic pulmonary disease, and an anomaly of the central nervous system on ultrasound). Although there was a decrease in the need for supplemental oxygen and positive blood cultures, the primary motive was not achieved. There was an elevated risk of necrotizing enterocolitis when amoxiclav (amoxicillin + clavulanic acid) was administered alone or in conjunction with erythromycin [28]. An initial aggressive approach using intravenous antibiotics was undertaken in the NICHD study. Intravenous (IV) ampicillin (two grams every six hours) and erythromycin (250 milligrams every six hours) were given for a period of 48 hours, followed by a five-day oral administration of amoxicillin along with enteric-coated erythromycin to finish a seven-day antibiotic regimen. After completing seven days of antibiotic therapy, patients' chances of being undelivered increased by twofold. The increased delay persisted for up to three weeks after the medications were stopped. This therapy also showed a reduction in neonatal morbidity and mortality as well as a reduction in the incidence of amnionitis [6]. Broad-spectrum antibiotic treatment lengthens pregnancy, lowers maternal and newborn infections, and lowers morbidity associated with gestational age. While several antibiotic regimens have been shown to be beneficial, it is uncertain which is the best. According to the information currently available, a seven-day course of latency antibiotic therapy with a combination of intravenous ampicillin and erythromycin followed by oral amoxicillin and erythromycin is recommended during expectant management of women with preterm PROM who are at less than 34 weeks of gestation in order to reduce maternal and neonatal infections and gestational-age-dependent morbidity [24].

Importance of Corticosteroids in the Management of PROM

Corticosteroids decrease the occurrence of respiratory distress syndrome, intraventricular haemorrhage, and necrotizing enterocolitis if given to a woman with intact membranes in the case of preterm labour [4]. A similar benefit of corticosteroids can also be seen in preterm PROM with gestational ages between 24 and 34 weeks. If used after 34 weeks, there is no evidence of benefit. All patients with preterm PROM between 24-34 weeks of pregnancy should consider using corticosteroids to accelerate lung maturation. It has been hypothesised that the latency period is too brief for corticosteroid effects to bring about a change in newborn morbidity; however, there is no evidence to support this hypothesis. Rather, it has been suggested that most of these PPROM patients will continue to remain pregnant for a period of 48 hours, and hence the corticosteroid therapy will benefit them. Women with preterm PROM who are less than 34 weeks gestational age are at risk of premature birth within seven days, and those whose last treatment of prenatal corticosteroids was provided more than 14 days ago can be eligible for a single repeat course of corticosteroids. To get a rescue course, nonetheless, delivery shouldn't be delayed [24]. The use of steroids is thought to be associated with an increased occurrence of infection. However, the present evidence does not support this issue, and hence there is no linkage to increased or decreased infection [6].

Induction of Labour

Early induction of labour is suggested in term PROM cases to prevent the risks of mortality and morbidity. For this purpose, the most commonly used intravenous agent is oxytocin. Another substitute for oxytocin is misoprostol, which is easier to use and can be administered by various routes rather than IV [29]. If rupture of membranes occurs at more than or equal to 37 weeks, induction of labour is done. The preferred mode of delivery is vaginal since a caesarean section is associated with increased chances of postpartum endometritis. If PPROM occurs between 34 and 36 weeks + six days, induction of labour is done if there is chorioamnionitis, fetal distress, or there are high chances of cord prolapse. In chorioamnionitis, there is no role for expectant management, corticosteroids, or tocolytics [24].

Tocolytics

There is no evidence that giving tocolysis is beneficial to a newborn [30]. Prophylactic tocolysis was reported to temporarily lengthen latency in one trial. Latency was reduced in another trial when magnesium sulphate was provided [31]. In conclusion, tocolytic drugs have not been properly studied in combination with latency antibiotics and corticosteroids. However, they may cause the pregnancy to last longer and raise the risk of chorioamnionitis without showing any advantage to the mother or the newborn [24]. Unlike corticosteroids and antibiotics, tocolysis should only be used when there is a proven clinical advantage, such as in transporting a pregnant woman to a tertiary care centre with the availability of a neonatal intensive care unit. According to available evidence, magnesium sulphate given before an expected early premature birth lowers the chance of cerebral palsy in infants [32,33]. Physicians who choose to employ magnesium sulphate for foetal neuroprotection should set precise protocols for eligibility, therapy regimens, simultaneous tocolysis, and surveillance [34]. Four or six-gram bolus, along with a maintenance dose of one-two gram, was used in these studies for 12-24 hours of exposure. Although it must be noted that prenatal magnesium sulphate therapy in the clinical case of chorioamnionitis in preterm children was not able to provide neuroprotection as expected [35]. Some obstetricians have advocated and are now using tocolysis for 48 hours to allow time to inject steroids and accelerate foetal lung maturity. There is no definite evidence to support the efficacy of this treatment, and the patient should be well informed regarding the possible risks and adverse effects before using tocolytics.

Prediction and prevention

Preterm labour and rupture of the membranes continue to be major contributors to preterm birth. In order to have the best newborn outcomes, the time between the rupture of the membranes and delivery is crucial. Predicting the latency period is still a challenge in obstetric practise. Certain studies have established that women with cervical lengths less than/equal to 2.5 cm have a decreased latency period in comparison to females with cervical lengths more than 2.5cm [36]. Hence, a short cervical length, a previous history of preterm birth caused by PPROM, and a positive foetal fibronectin screening are factors that can predict PPROM. Women who have had previous preterm deliveries should be advised that short interpregnancy intervals, particularly those less than six months, may lead to unfavourable pregnancy outcomes [37]. Women with a singleton pregnancy and a prior history of preterm birth should be given progesterone supplementation from 16-20 weeks until 36 weeks to lower the risk of recurrent spontaneous preterm birth. Assessment of cervical length must also be done for these women between 16 and 24 weeks, and those with a length less than or equal to 2.5 cm should undergo cervical cerclage before 24 weeks. There is no role for bed rest, nutrient supplementation, or empirical antibiotics in prevention [24].

## Conclusions

Pre-labour rupture of membranes is one of the major contributors to perinatal mortality and morbidity in pregnant women. Out of all the preterm births, almost 40-50% are linked to PROM, in which the prognosis depends upon gestational age and mode of delivery. A timely and precise diagnosis of PROM is essential for a successful pregnancy. The traditional and old clinical tests, such as nitrazine paper tests, pooling, ferning, and amnio dye tests, are eventually being replaced by the comparatively newer and more reliable AmniSure and Actim tests. Admission to the hospital, amniotic fluid sampling to rule out the presence of infection, expectant management, antenatal corticosteroids along with broad-spectrum antibiotic therapy only if indicated, and delivery once a desirable age of gestation is reached are all options once the diagnosis is confirmed. The primary motive is to ameliorate the perinatal outcome and decrease newborn morbidity and mortality. This can only be achieved if clinicians develop a clear understanding of the evaluation and management of PROM.
